# BioDSNN: a dual-stream neural network with hybrid biological knowledge integration for multi-gene perturbation response prediction

**DOI:** 10.1093/bib/bbae617

**Published:** 2024-11-25

**Authors:** Yuejun Tan, Linhai Xie, Hong Yang, Qingyuan Zhang, Jinyuan Luo, Yanchun Zhang

**Affiliations:** The Cyberspace Institute of Advanced Technology, Guangzhou University, Guangzhou 510000, China; School of Computer Science and Technology, Zhejiang Normal University, Jinhua 321000, China; State Key Laboratory of Proteomics, National Center for Protein Sciences (Beijing), Beijing 100000, China; International Academy of Phronesis Medicine, Guangzhou 510000, China; The Cyberspace Institute of Advanced Technology, Guangzhou University, Guangzhou 510000, China; International Academy of Phronesis Medicine, Guangzhou 510000, China; The Cyberspace Institute of Advanced Technology, Guangzhou University, Guangzhou 510000, China; School of Computer Science and Technology, Zhejiang Normal University, Jinhua 321000, China

**Keywords:** genetic perturbation predict, biological knowledge, masked attention, variational autoencoder

## Abstract

Studying the outcomes of genetic perturbation based on single-cell RNA-seq data is crucial for understanding genetic regulation of cells. However, the high cost of cellular experiments and single-cell sequencing restrict us from measuring the full combination space of genetic perturbations and cell types. Consequently, a bunch of computational models have been proposed to predict unseen combinations based on existing data. Among them, generative models, e.g. variational autoencoder and diffusion models, have the superiority in capturing the perturbed data distribution, but lack a biologically understandable foundation for generalization. On the other side of the spectrum, Gene Regulation Networks or gene pathway knowledge have been exploited for more reasonable generalization enhancement. Unfortunately, they do not reach a balanced processing of the two data modalities, leading to a degraded fitting ability. Hence, we propose a dual-stream architecture. Before the information from two modalities are merged, the sequencing data are learned with a generative model while three types of knowledge data are comprehensively processed with graph networks and a masked transformer, enforcing a deep understanding of single-modality data, respectively. The benchmark results show an approximate 20% reduction in terms of mean squared error, proving the effectiveness of the model.

## Introduction

After centuries of investigation and revelation in biology, human beings are still haunted with a fundamental problem about how a cell, the minimal unit of life, works in our body. Besides the static portraits of the molecular distribution inside, such as the genome, transcriptome, proteome, and metabolome, a more complex attribute of a cell is how these molecules interact with each other, which gives rise to the dynamic and diverse functions of cells. Currently, genetic perturbation is a crucial method to study the genetic interactions and their impact on cellular functions [1] and the resulted discoveries are serving as new theoretical foundations for biotechnology and disease treatment [2].

Thanks to the rapid development of Single-cell RNA sequencing (scRNA-seq) methods [3, 4], such as high-throughput perturbational screens [5] and CRISPR-based perturbational screens [6], which have made notable advancements and gained widespread adoption [7], scientists can quickly sample the downstream outcomes on cell state experimentally[8]. However, due to the vast number of human cells and the explosive combination of perturbations, studying cell responses under all possible perturbations purely through lab experiments seems to be impossible in terms of time and manpower costs.

Fortunately, because of this immense sample size compared to bulk data, single-cell data are well suited for modeling by deep learning techniques in various kinds of tasks, e.g. segmentation [9, 10], clustering [11], and drug effect prediction [12]. Among them, modeling cellular response by predicting gene expressions after perturbation are also of significance, including both genetic perturbation (knockout or overexpression) and chemical perturbations [13]. For instance, scGen [14] employs a variational autoencoder (VAE) to predict the perturbation response of new cell types by estimating the perturbation in latent space. scVAEDer [15] proposes a scalable deep-learning model that combines the power of VAEs [16] and deep diffusion models [17] to learn a meaningful representation that captures both global semantics and local variations in the data. scPreGAN [18] integrates autoencoder and Generative Adversarial Network [19] to predict the response of single-cell expression to perturbation. And GenKI [20] adapts a Variational Graph Autoencoder model to predict shifting patterns in gene regulation caused by gene Knock Out(KO) perturbation in an unsupervised manner.

Although generative models are good at capturing the underlying distribution of gene expressions; unfortunately, these pure data-driven models lack a reliable generalization ability neither intuitively nor empirically. On the one hand, we can not be informed from the black-box model that whether it has learned reasonable genetic relationships, which is a favored biological foundation of the prediction. On the other hand, the amount of tested unseen perturbations is relatively tiny compared to the entire possible space.

Since a reliable evaluation on massive combinations of possible perturbations is costly, many researchers have turned to intuitively more generalizable approaches, i.e. forcing the model to predict based on biological knowledge. Kenji *et al*. [21] utilizes Gene Regulatory Networks (GRNs) [22] inferred from data to model perturbation response, but the fitting ability of the model is limited with a simple architecture. Wu *et al*. [23] improve the prediction performance by integrating GRNs into a deep variational causal inference framework. However, it could only predict the outcomes of single perturbations, whereas multiple perturbations are more representative of real-world experiments. Roohani *et al*. [24] propose GEARS to infer multi-gene perturbation response by directly stacking the unperturbed gene expressions to postperturbation embeddings which are learned from Gene Ontology (GO) and gene co-expressions respectively through Graph Neural Networks(GNNs). However, it integrates information among genes after the GNN only through a few dense layers, and then directly adds the unperturbed gene expressions to the graph processed perturbation gene embeddings to obtain the final predictions, which limits the model’s ability to capture the latent distribution and uncertainty of gene expressions, reducing its generalization capability.

To overcome the aforementioned challenges and improve model performance, we propose a novel Biological knowledge guided Dual-Stream Neural Network (BioDSNN) model to predict multi-gene perturbation response. In contrast to GEARS, our model introduces a data-driven stream that leverages generative models to capture the intrinsic distribution of gene expressions within cells. Simultaneously, the knowledge-driven stream incorporates three distinct types of biological information to guide the model, and an attention encoder is utilized to enhance the understanding of the relationships between genes and perturbations. The proposed model has demonstrated a higher level of accuracy in predicting the outcomes of perturbations in individual genes or gene combinations, even without prior experimental perturbation data.

## Materials and methods

### Problem Formulation and Model Framework

This work aims to enhance the performance of novel perturbation response prediction. Given a perturbation dataset of $n$ cells $\mathcal{D}=\left \{\left (g^{i},p^{i}\right )\right \}^{n}_{i=1}$, where $g^{i}\in \mathbb{R}^{m}$ is the gene expressions vector of cell $i$ with $m$ genes, and $p^{i}=\left (P^{i}_{1},...,P^{i}_{z}\right )$ is the set of perturbations of size $z$ performed on cell $i$. The objective of BioDSNN is to predict the perturbed gene expressions outcome, which is a new gene expressions vector $g$.

For the data-driven stream, BioDSNN first collects the unperturbed gene expressions data and initialize a gene expressions matrix $X\in \mathbb{R}^{n\times{m}}$, where $n$ is the number of cell samples and $m$ is the number of genes. The matrix is subsequently regenerated by reparameterizing it from the latent space through a VAE flow.

In the knowledge-driven stream, gene and perturbation embeddings are initialized with dimensions ${n}\times{m}\times{d}$ and ${z}\times{d}$, respectively. A GNN encoder, parameterized by $\theta _{g}$, augments each gene embedding by integrating information from $G_{gene}$, while another GNN encoder, $\theta _{p}$, enhances perturbation embeddings using $G_{pert}$. BioDSNN then combines specific perturbation embedding with each of the gene embeddings, and the resulting postperturbation gene embeddings are processed through a transformer-like masked encoder for further refinement. Since the postperturbation gene embeddings need to be added to the gene expressions matrix in the data-driven stream, BioDSNN sums over the last dimension of the postperturbation gene embeddings, reshaping them into ${n}\times{m}$. Finally, the postperturbation gene embeddings is added to the ${n}\times{m}$ gene expressions matrix to generate the final gene-specific predictions.

We adopt the gene relationship graph and perturbation relationship graph from GEARS. Following this, GEARS employs a simple cross-gene MLP layer to process the postperturbation gene embeddings. In contrast, we incorporate the Reactome pathway database into a masked attention encoder. This encoder captures the perturbation and its relationships to other genes, generating an augmented Postperturbation gene embeddings. This approach enables the model to better capture complex features and enhance its representational capacity. Additionally, while GEARS directly adds the unperturbed gene expressions to the postperturbation gene embeddings, we utilize a VAE during this process to regenerate the unperturbed gene expressions, which helps capture the appropriate data distribution and the uncertainty more effectively.

**Figure 1:**
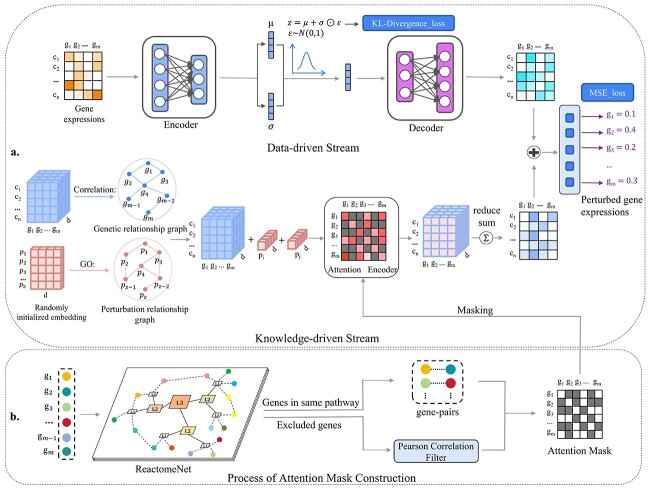
Illustration of an interpretable dual-stream framework. (a) The main architecture of BioDSNN consists of two streams. In the data-driven stream, the input is the unperturbed gene expressions, which are encoded and resampled during training and then regenerated for the final prediction. In the knowledge-driven stream, gene and perturbation embeddings are initialized and fed into a GNN, enhanced by gene correlations and perturbation relationships. The specific perturbation embeddings are then combined with each gene embedding. Next, the postperturbation gene embeddings are processed by a transformer-like masked encoder, with the mask constraining attention to relevant genes. Since the postperturbation gene embeddings need to be added to the gene expressions matrix in the data-driven stream, BioDSNN sums over the last dimension of the postperturbation gene embeddings, reshaping them into ${n}\times{m}$. The postperturbation gene embeddings then combine with the regenerated gene expressions matrix to produce the final gene-specific predictions. (b) The attention mask is constructed by feeding the gene set into ReactomeNet to identify gene pairs in the same pathway. For these pairs, the corresponding positions in the mask matrix are set to zero, while all other positions are set to negative infinity, preventing attention computation in the Knowledge-driven stream’s Attention Encoder because of the softmax function. For genes not in the database, Pearson correlations with other genes are calculated, and only correlations above a certain threshold have their positions set to zero.

Our methods overview is shown in Fig. 1.

### Data-driven Stream

Compared to GEARS, our model incorporates a novel Data-driven Stream. In GEARS, after obtaining the augmented postperturbation gene embeddings through a GNN, it is directly added to the gene expressions in the unperturbed state. Instead, we aim to utilize a VAE to capture the uncertainty and variability inherent in the gene expressions data, which is demonstrated as effective in subsequent experiments.

We apply it to obtain representative embedding for raw gene expressions, and then only regenarate the gene expressions that are originally non-zero values in the control group.

VAE measures the divergence between the learned distribution of the latent variables (given the raw gene expressions data) and a prior distribution, typically a Gaussian prior distribution N(0,1). Specifically, VAE maximizes the lower bound on the evidence, known as the Evidence Lower Bound(ELBO), as the marginal likelihood is intractable, the variational lower boundary of the marginal likelihood of input data is the objective function of VAE. The marginal likelihood is obtained by summing of the marginal likelihood of distinct data points as below:


(1) \begin{align*}& \begin{aligned} log\,p_{\theta} \left ({x^{\left ({1 }\right)},\ldots,x^{\left ({N }\right)}}\right)=\sum \limits_{i=1}^{N} {log\,p_{\theta }\left ({x^{\left ({i }\right)}}\right)} \end{aligned}\end{align*}

The marginal likelihood of distinct data points can be reformulated as follows:


(2) \begin{align*}\log p_{\theta}(x^{(i)}) &\geq \mathcal{L}_{\text{ELBO}}(\theta, \phi; x^{(i)})\nonumber \\&= -D_{KL}(q_{\phi}(z \mid x) \| p_{\theta}(z))\nonumber \\&\quad + \mathbb{E}_{q_{\phi}(z \mid x^{(i)})}[\log p_{\theta}(x \mid z)]\end{align*}

(3) \begin{equation*}\mathcal{L}_{\text{ELBO}} = \mathbb{E}_{q_{\phi}(z \mid x^{(i)})}\left[ -\log q_{\phi}(z \mid x) + \log p_{\theta}(x \mid z) \right]\end{equation*}

The likelihood of the input data $x$, given the latent variable $z$, is represented as $p_\theta (x|z)$.


(4) \begin{align*}& L_{KL} = D_{KL} = \frac{1}{2} \sum_{j=1}^{d} \left( 1 + \log(\sigma_{j}^{2}) - \mu_{j}^{2} - \sigma_{j}^{2} \right)\end{align*}

$D_{KL}$ is the Kullback–Leibler divergence between the approximate posterior and the prior of the latent variable $z$. $q_{\phi }(z \mid x)$ is the approximate posterior distribution over the latent variable $ z $ with mean $ \mu $ and variance $ \sigma ^{2} $. $p_{\theta }(z)$ is the prior distribution over the latent variable $ z $, typically assumed to be $ \mathcal{N}(0, I) $. $ \mu _{j} $ and $ \sigma _{j} $ are the mean and standard deviation of the $ j $th dimension of the latent variable $ z $ in the approximate posterior. $ d $ is the dimensionality of the latent variable $ z $.

The output of the decoder is a new representation of gene expressions data. And the scRNA-seq data are characterized by its high sparsity, with critical cell information primarily concentrated in the non-zero expression values [25]. In our approach, we retain the original zero values in the gene expressions data and regenerate only the non-zero portions. This method, though simple, effectively addresses the long-tail effect in genome-wide expression predictions, as each cell type transcribes only a small fraction of genes to fulfill its specific functions.

### Knowledge-driven Stream

To improve performance, we integrate a transformer-like encoder into our knowledge-driven stream, incorporating three categories of biological prior knowledge.

#### Applying co-expression patterns for identify genetic relationship

Genetic relationship includes various kinds of interactions and regulations. A common method to capture parts of them is to identify gene pairs with strong co-expression patterns [26]. Therefore, in our model, we construct a gene co-expression graph $G_{gene}$ by computing the Pearson correlation coefficients $P_{u,v}$ between genes $u,v$ in the datasets.

#### Utilizing GO for biological insights

We adopt the approach of handling GO [27] from GEARS. More specifically, GO pathways are utilized to measure the functional similatiry between genes. For instance, $N_{u}$ is the set of pathways of gene $u$, then Jaccard index between a pair of genes $u,v$ is computed as $J_{u,v}=\frac{\vert N_{u}\cap N_{v}\vert }{\vert N_{u}\cup N_{v}\vert }$, which measures the fraction of shared pathways between them. For each gene, we select top $H_{p}ert$ gene with the highest $J_{i}ndex$ to construct perturbation similarity graph $G_{pert}$. After that, all initialized possible gene perturbations embeddings will be fed into a simplified graph convolutional networks [28] to augment. In this way, neighboring perturbations’ information could be integrated in every perturbation embedding.

#### Leveraging Reactome for pathway analysis

**Figure 2:**
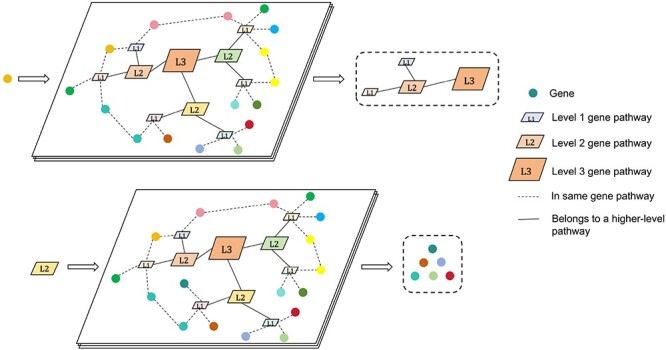
Illustrates the main function of ReactomeNet.

In this paper, we incorporate Reactome[29], a new source of biological prior knowledge not utilized in GEARS. Reactome provides a comprehensive framework for mapping and visualizing molecular interactions and pathways, allowing for a deeper understanding of complex biological processes. We obtain hierarchical pathway information for Homo sapiens from the database [29] and implement a tool called ReactomeNet to construct a directed graph from raw data. It provides all pathway levels associated with any gene or gene list. ReactomeNet can also retrieve all genes associated with any pathway from the database. Figure 2 illustrates ReactomeNet. The upper section demonstrates that when a single gene is input, ReactomeNet retrieves all the pathways associated with that gene at various levels. The lower section shows that when a pathway is input, the network returns the set of genes involved in that pathway. The peripheral colored circles represent genes, all of which belong to the L1 level pathways. L3 represents the highest level of pathways, with L2 and L1 being lower-level pathways. Additionally, L2 is a sub-pathway of L3, while L1 is a sub-pathway of L2.

In Fig. 1(b), the attention mask is constructed by feeding the gene set into ReactomeNet to identify gene pairs in the same pathway. For these pairs, the corresponding positions in the mask matrix are set to zero, while all other positions are set to negative infinity (represented as gray blocks), preventing attention computation in the Knowledge-driven stream’s Attention Encoder because of the softmax function.

#### Masked attention

Building on GEARS’ approach of embedding prior knowledge, we have introduced a self-attention mechanism through a transformer-like architecture. This approach enhances both the model’s fitting ability and interpretability. Our trials show significant performance improvements, mainly because of the enhanced fitting capability of the self-attention mechanism and the more focused attention space, which is constrained by pathway masks when modeling perturbation effects.

More concretely, this module uses the self attention mechanism and the core part is scaled dot-product attention. Its calculation formula can be described as follows:


(5) \begin{align*}& \begin{aligned} Attention(Q,K,V) = Softmax\left(\frac{QK^{T}}{\sqrt{d_{k}}}\right)V \end{aligned}\end{align*}

where $d_{k}$ is the dimension of matrix $Q$ and $K$. Self attention first performs $h$ times linear transformation on $Q$, $K$, and $V$ respectively, where the parameter matrix of each linear transformation is different. We add the mask to the scaled dot product $QK^{T}$ before computing the softmax function. Typically, the values of the mask are set to a very large negative number such as $-\infty $. This setting ensures that the weights associated with these positions are close to zero after passing through the softmax function, effectively prevents the model from attending to not relevant areas, which do not contain meaningful information. In this way, we could obtain high-level gene representations $I \in \mathbb{R}^{c\times{m}\times{d}}$ via the self attention mechanism.


(6) \begin{align*}& \begin{aligned} I \in \mathbb{R}^{c\times{m}\times{d}} = Trm(f_{Q}(I),f_{K}(I),f_{V}(I)) \end{aligned}\end{align*}

where $c$ is the num of cells, $m$ is the num of genes, $d$ means the dimension of embeddings, and $f_{Q}, f_{K}, f_{V}$ are the project functions. $Trm$ denotes the transformer block.

### Autofocus and direction loss

We employ an autofocus loss, which mainly inherits from mean squared error (MSE) loss. It could automatically give a higher weight to differentially expressed (DE) genes by elevating the exponent of the error, and it is defined as follows:


(7) \begin{align*}& \begin{aligned} L_{autofocus} = \frac{1}{T} \sum_{k=1}^{T} \frac{1}{T_{k}} \sum_{l=1}^{T_{k}} \frac{1}{K} \sum_{u=1}^{K}{\left(g_{u}-\hat{g_{u}}\right)}^{(2+y)} \end{aligned},\end{align*}

Given a minibatch of $T$ perturbations, each perturbation $k$ has $T_{k}$ cells and each cell has $K$ genes with predicted perturbed gene expressions $g_{u}$ and true expression $\hat{g_{u}}$.

Due to the $L_{autofocus}$ is insensitive to directionality, an additional direction-aware loss is incorporated:


(8) \begin{align*} & L_{direction} = \frac{1}{T} \sum_{k=1}^{T} \frac{1}{T_{k}} \sum_{l=1}^{T_{k}} \frac{1}{G} \sum_{u=1}^{K}{h(u)}^{2} \end{align*}

(9) \begin{align*}\qquad\ \ h(u) &= \operatorname{sign}(g_u - g_u^{\text{ctrl}}) - \operatorname{sign}(\hat{g_u} - g_u^{\text{ctrl}})\end{align*}

We summarize above loss functions and get the overall joint prediction loss function as shown below:


(10) \begin{align*}& \begin{aligned} L = L_{KL} + \beta L_{autofocus} + \lambda L_{direction} \end{aligned},\end{align*}

where $\beta $ adjusts the weights for the autofocus loss and $\lambda $ adjusts the weights for the directionality loss.

## Results

We now present our experiments to validate the effectiveness of the proposed framework.

### Datasets

To predict transcriptional outcomes caused by perturbations, we select three publicly available perturbation sets consisting of single-gene or multiple genes. For single-gene, We use data from two different genetic perturbation screens consisting of 1543 (RPE1 cells) and 1092 (K562 cells) perturbations, respectively, with each measuring over 170 000 cells (Replogle *et al*.[30]). In addition, the evaluated data set (Norman *et al*.[31]) contains 131 two-gene perturbations, then we could assess the performance across different perturbation levels. The summary of the datasets is shown in Table 1.

**Table 1:** Composition of the datasets

Datasets	Cell_samples	Genes	Perturbations
K562	162 751	5000	1092
RPE1	162 733	5000	1543
Norman	91 205	5045	131 (2-gene)

### Experimental settings

In our experiments, the BioDSNN model was trained with data split into training(70%), validation(20%), and test sets(10%) to capture single and combinatorial perturbations. A hidden size of 64 was used throughout, with one GNN layer each for gene co-expression and gene ontology graphs. A Transformer Encoder layer replaced the cross-gene module to compute attention between nodes, utilizing a hidden dimension of 64, a 512-dimensional feedforward network.

To regularize gene expressions, a VAE with a latent dimension of 16 and an intermediate size of 32 was applied before perturbation, minimizing both reconstruction loss and KL divergence. The model was trained for 20 epochs with the Adam optimizer (lr=0.001, weight decay=5e-4), and a step learning rate scheduler (step size=1, decay=0.5). The loss function combined MSE and directional loss to ensure consistent gene expressions changes relative to the control. Hyperparameters were tuned on validation performance for optimal accuracy and interpretability.




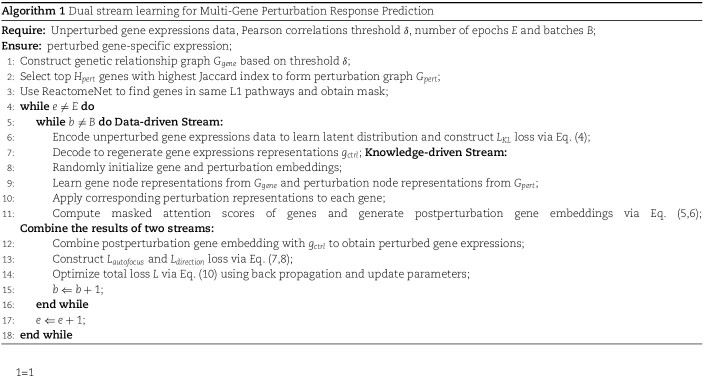



### Predicting gene perturbation outcomes

To evaluate the differences in gene expressions of single cells before and after perturbation, we calculate the MSE for each gene between the control and experimental groups. As the vast majority of genes do not show substantial variation between unperturbed and perturbed states, we only consider the top 20 most DE genes.

For each gene $g$, we compute its average expression levels in the control group and the experimental group, denoted as $\bar{x}^{(c)}_{g}$ and $\bar{x}^{(e)}_{g}$, the formula is as follows:


(11) \begin{align*}& \begin{aligned} \text{MSE}_{g} = \frac{1}{N} \sum_{i=1}^{N} \Big(\bar{x}_{g,i}^{(c)} - \bar{x}_{g,i}^{(e)}\Big)^{2} \end{aligned}, \end{align*}

where $N$ is the number of samples, $\bar{x}_{g,i}^{(c)}$ and $\bar{x}_{g,i}^{(e)}$ are the expression levels of gene $g$ in the $i$th sample.

**Figure 3:**
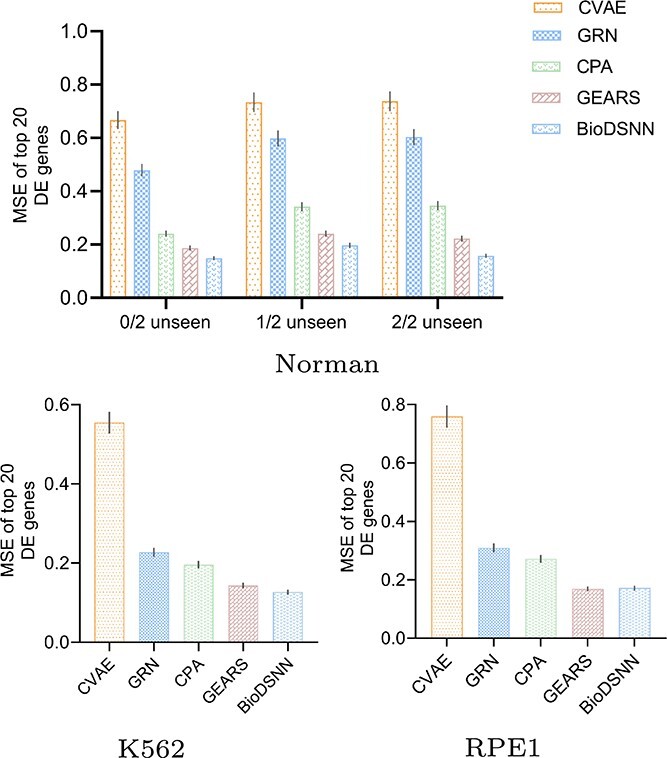
MSE for gene perturbation response prediction.

Figure 3 illustrates the MSE metric in predicted postperturbation gene expressions for two-gene and single-gene perturbations, where the 20 most DE genes are considered. Figure 3(a) denotes two-gene perturbation prediction, where the $x$-axis progressively denotes the number of the perturbed targets that have not been seen in the training set, with an increasing difficulty for prediction. ‘0/2 unseen’, ‘1/2 unseen’, and ‘2/2 unseen’ denotes two, one or zero perturbed targets are seen in the training set, respectively. (b) and (c) denote single perturbation prediction task. All the black markers highlight the mean and error bars corresponding to 95% confidence intervals.

The results in Fig. 3 demonstrate that our BioDSNN model outperforms almost all baselines in the gene perturbation prediction task, reflecting the model’s strong ability to capture regulatory relationships. Specifically, BioDSNN shows approximately a 20% improvement over GEARS across all datasets. This improvement is due to modifications to the dual-stream network, which were each validated for effectiveness through ablation experiments, ensuring that the model learns from data while integrating valuable biological insights. The application of transformer-like encoder enables the model to focus on the most relevant features, dynamically adjusting the importance of different genes and perturbations. Meanwhile, the use of masked attention ensures that the model can handle complex dependencies and interactions within the gene expressions data, leading to more accurate predictions.

### Comparing the proportion of accurately predicted genes

**Figure 4:**
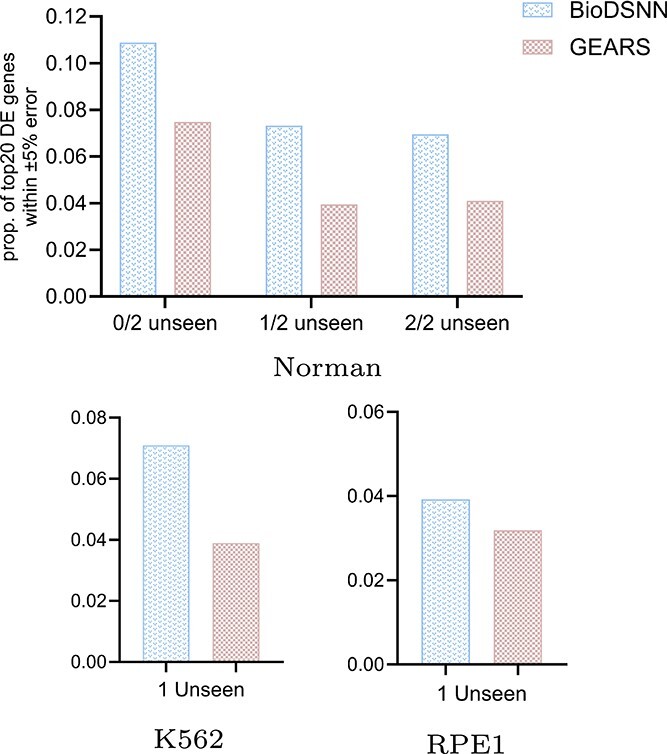
Proportion of genes with predicted vs. true expression values within $\pm 5\%$ error among top 20 DE genes.

We conduct analysis to determine the proportion of predicted values that fell within $\pm 5\%$ of the true mean expression value for the top 20 DE genes. As illustrated in Fig. 4, the results demonstrate that the BioDSNN model consistently achieves a higher percentage of predictions within this error range compared to the baseline models across three distinct datasets. This enhanced performance highlights our model’s accuracy in estimating gene expressions, and demonstrating that it provides a more reasonable distribution of perturbed gene expressions values.

The elevated proportion of predictions within $\pm 5\%$ of the true values indicates that BioDSNN model effectively captures the nuances of gene expressions, providing a more precise representation of perturbed expression changes. The robustness of BioDSNN model across diverse datasets further reinforces its reliability and generalizability in gene expressions prediction tasks.

This performance underscores the model’s capability to consistently deliver accurate predictions, which is critical for applications requiring precise gene expressions data.

### Assessing the linear correlation of gene expressions

In addition to the MSE, we utilize the Pearson Correlation Coefficient (PCC) to assess the consistency in single-cell gene expressions before and after perturbation, regardless of the scale of the expression values. This dual metric approach facilitates the elucidation of linear relationships and relative changes in gene expressions, providing a comprehensive view of how perturbations affect gene expressions profiles. By examining both MSE and Pearson correlation, we capture not only the magnitude of expression changes but also the consistency and directionality of these changes across individual cells. This method allows for a more nuanced understanding of the model’s performance, highlighting both the accuracy of predicted expression values and correlation between predicted and observed changes.

**Figure 5:**
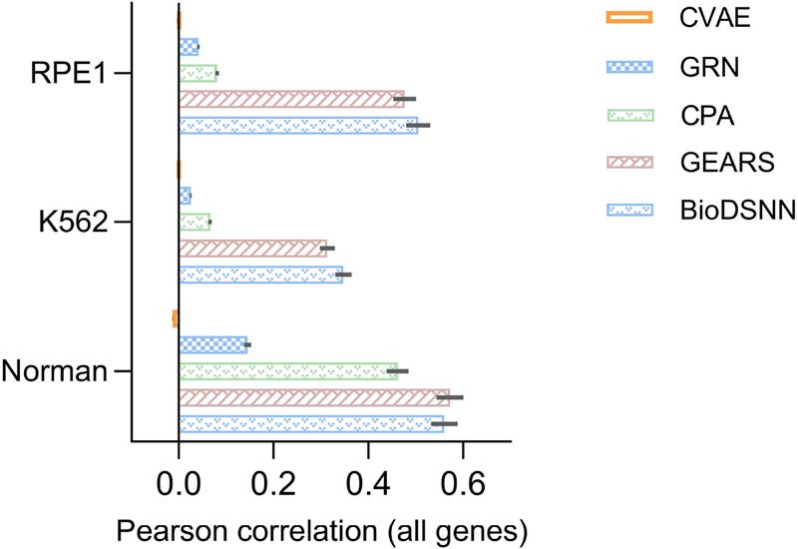
Pearson correlation between mean perturbed differential gene expressions over control and true values across all genes.

As illustrated in Fig. 5, our model exhibits superior performance in the K562 and RPE1 datasets, achieving the highest accuracy in predicting gene expressions changes. This performance surpasses that of baseline models, underscoring the model’s efficacy in capturing gene expressions dynamics. For Norman dataset, the performance of our model is comparable to the GEARS model, indicating competitive predictive accuracy across varying experimental contexts.

### Evaluating gene interaction effects

We screen all two-gene perturbations from the test set in Norman dataset, and compute the Magnitude Scores to estimate the genetic interaction effects between them [32]. We predict the gene expressions influenced by each two-gene perturbation combination and to separately predict the gene expressions resulting from individual perturbations within the combinations. Then we employ Theil–Sen Regressor to investigate whether the interaction effects of individual gene perturbations affect final gene expressions, which is defined by


(12) \begin{align*} & \begin{aligned}\quad \mathbf{y} = \mathbf{X} \boldsymbol{\beta} + \epsilon \end{aligned}, \end{align*}

(13) \begin{align*}\boldsymbol{\beta} &= \begin{bmatrix}c_1 \\c_2\end{bmatrix}\end{align*}

Where $\mathbf{X}$ is the matrix of two single perturbation expressions with dimensions ${m}\times{2}$, m is the number of genes, $\boldsymbol{\beta }$ is the vector of regression coefficients and $\epsilon $ is the error term. The coefficients $c_{1}$ and $c_{2}$ are extracted from the fitted model. The magnitude score is the Euclidean distance between $c_{1}$ and $c_{2}$, calculated as


(14) \begin{align*}& \text{mag} = \sqrt{c_{1}^{2} + c_{2}^{2}} \end{align*}

**Figure 6:**
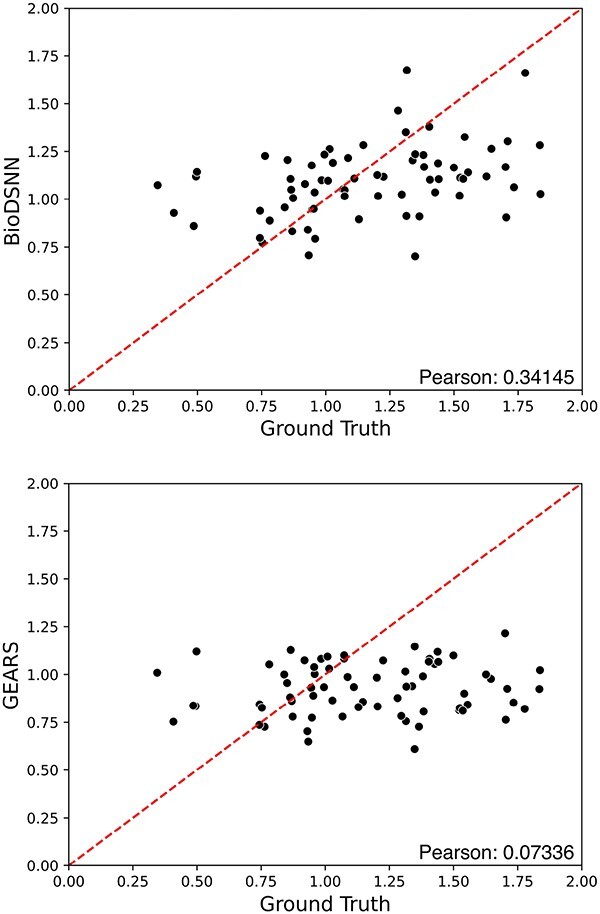
Magnitude scores computed for all test perturbing combinations on the Norman dataset.

The magnitude scores represent the overall strength of the regression coefficients. As depicted in Fig. 6, each dot corresponds to a specific perturbation combination. The $y$-axis displays magnitude scores derived from model’s predictions, while the $x$-axis shows the ground truth magnitude scores calculated from actual perturbed gene expressions data. The results indicate that BioDSNN model exhibits a closer alignment with the ground truth compared to other models.

**Figure 7:**
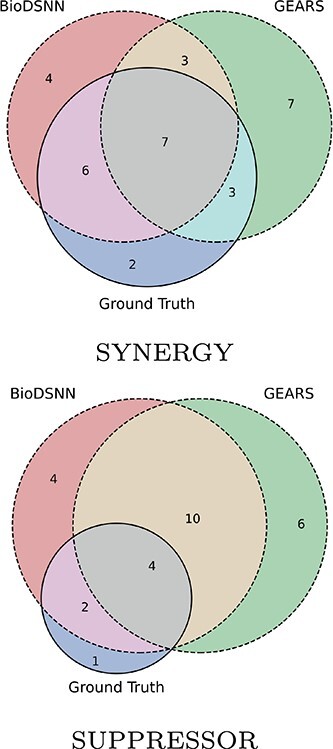
Top 20 perturbations with synergistic and suppressor gene interaction types identified using BioDSNN and baseline methods.

Additionally, we compute the PCC between the predicted and ground truth genetic interaction (GI) magnitude scores for all two-gene perturbations in test set. The result reveals that BioDSNN achieved a significantly higher PCC than the GEARS model. This enhanced correlation suggests that BioDSNN provides a more accurate representation of genetic interactions, effectively capturing the underlying patterns of data.

**Figure 8:**
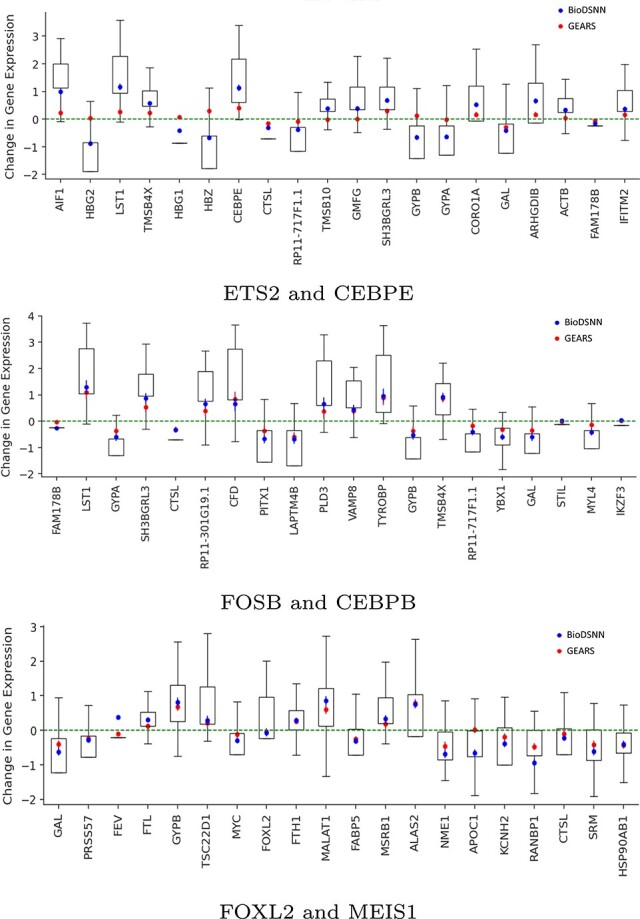
Change in gene expressions after perturbing two genes.

We also design to classify two-gene perturbations into distinct GI types. To this end, we identify synergy and suppressor GI types using the magnitude score. We rank the two-gene perturbations based on the predicted magnitude scores, and classify top 20 perturbations as potential synergy GI types and bottom 20 perturbations as potential suppressor GI types. Subsequently, these classifications are compared with the perturbation combinations corresponding to synergy and suppressor GI types in the ground truth dataset. As illustrated in Fig. 7, BioDSNN outperforms GEARS by detecting more two-gene perturbations that align with the true GI types.

The results above underscore the superior performance of BioDSNN in predicting genetic interaction magnitudes. The improved correlation with the ground truth data highlights the model’s robustness and reliability in capturing the complexities of gene perturbations.

### Predicting perturbation trends and magnitudes

In the realm of disease research, alterations in gene expressions can provide valuable insights into potential biomarkers for diagnosis and prognosis. Genes exhibiting significant changes in expression levels may also emerge as promising targets for therapeutic interventions and drug development.

To assess the individual gene responses to perturbations, we predict the outcomes of perturbing three types of gene combinations, each representing different levels of training set visibility: two genes seen, one gene seen, and zero genes seen during training. Specifically, for the ETS2 and CEBPE combination, both genes are seen during training. For the FOSB and CEBPB combination, CEBPB is not experimentally perturbed during training. In the case of the FOXL2 and MEIS1 combination, neither gene is seen during training.

As illustrated in Fig. 8, our model effectively capture both the trend and magnitude of perturbation across all 20 DE genes. The blue symbols represent the mean changes in gene expressions predicted by BioDSNN, while the red symbols correspond to predictions made by GEARS. The green dotted line indicates the mean gene expressions under unperturbed control conditions. The whiskers on the plot denote final data points within 1.5 times of the interquartile range, providing an additional measure of variability in gene expressions data.

### Ablation study

To assess the effectiveness of the introduced dual-stream framework, we conducted an ablation study across all datasets, systematically removing each module to observe its impact on performance. By comparing these results, we isolated each module’s contribution to the model’s overall effectiveness. Tables 2 and 3 detail the outcomes, presenting performance metrics for each dataset. The tables highlight the added VAE and masked attention modules’ effectiveness, underscoring their significance in enhancing the model’s predictive capabilities.

**Table 2:** MSE results of ablation experiments

Models	Norman$_{seen2/2}$	Norman$_{seen1/2}$	Norman$_{seen0/2}$	K562$_{single}$	RPE1$_{single}$
CVAE	0.6675	0.7334	0.7377	0.5547	0.7590
GRN	0.4790	0.5982	0.6034	0.2273	0.3096
CPA	0.2405	0.3417	0.3561	0.1959	0.2714
GEARS	0.1871	0.2406	0.2227	0.1435	0.1729
Ours$_{noVAE}$	0.1648	0.2030	0.1727	0.1355	**0.1607**
Ours$_{noAttention}$	0.1843	0.2188	0.1733	0.1349	0.1879
Ours$_{noMask}$	0.1587	0.1998	0.1682	0.1304	0.1826
Ours	**0.1491**	**0.1971**	**0.1576**	**0.1267**	0.1764

**Table 3:** Pearson correlation of ablation experiments

Datasets	CVAE	GRN	CPA	GEARS	Ours$_{noVAE}$	Ours$_{noAttention} $	Ours$_{noMask}$	Ours
Norman	−0.0134	0.1453	0.4622	0.5722	**0.6231**	0.5699	0.5471	0.5545
K562	0.0046	0.0254	0.0659	0.3135	0.3304	0.3289	0.3311	**0.3474**
RPE1	−0.0043	0.0416	0.0807	0.4769	0.4876	0.4912	0.4983	**0.5054**

## Conclusion

We propose a novel deep neural network-based model called BioDSNN, which accurately predicts single-cell transcriptional responses to both single and multigene perturbations. The model incorporates a VAE to learn latent representations, a feature absent in GEARS. Additionally, BioDSNN employs a masked attention encoder to capture interactions among closely linked genes, with the mask derived from ReactomeNet, whereas GEARS uses a few dense layers for information integration. BioDSNN excels at predicting perturbation outcomes, effectively capturing both trends and magnitudes. It addresses the generalization and fitting issues of GEARS, achieving a leading performance across multiple tasks, with approximately a 20% reduction in MSE, demonstrating the model’s effectiveness. The efficacy of BioDSNN, however, is highly dependent on the quality of the training data and the extent of prior knowledge available. It is crucial that BioDSNN is trained on data derived from the same cell type as those targeted in subsequent predictions to ensure accuracy. This specificity underscores the importance of precise and relevant data collection in the initial stages of model development.

Our future work will focus on exploring additional perturbation combinations to enhance the understanding of genetic interactions. We also anticipate broadening the applicability of BioDSNN to achieve a more generalizable tool that can be utilized across various cell types and experimental conditions. This work will involve rigorous testing and validation to enhance the model’s versatility and predictive power, potentially accelerating the process of perturbational screening[33] and its applications in biomedical research.

**Key Points**

- Deep neural networks have been broadly applied to predict genetic perturbations based on single cell RNA-seq data. To enhance their generalization ability on unseen cell types and perturbations, biological knowledges have been exploited in several models. However, how to integrate different kinds of prior knowledge and how to merge them with genetic data need further investigation.
- We propose a dual-stream framework called BioDSNN to balance the learning of data distribution and prior knowledge, ensuring that each of them is fully exploited through specifically tailored architectures before integration.
- We explore the integration of GNNs and transformers to incorporate gene correlations, Gene Ontology annotations, and Reactome pathway information, enabling a deeper utilization of human biological knowledge and providing our model with stronger fitting capacity and generalizability.
- We benchmark our model on three distinct real-world datasets, achieving outstanding performance compared to existing methods. Specifically, our model demonstrates an approximate 20% reduction in terms of mean squared error (MSE).

## Data Availability

The implementation of this work is available at https://github.com/yu3jun/BioDSNN.

## References

[1] Jaitin DA , Weiner A, Yofe I. et al. Dissecting immune circuits by linking CRISPR-pooled screens with single-cell RNA-seq. Cell 2016;167:1883–1896.e15. 10.1016/j.cell.2016.11.039. 27984734

[2] Katti A , Diaz BJ, Caragine CM. et al. CRISPR in cancer biology and therapy. Nat Rev Cancer 2022;22:259–79. 10.1038/s41568-022-00441-w. 35194172

[3] Adamson B , Norman TM, Jost M. et al. A multiplexed single-cell CRISPR screening platform enables systematic dissection of the unfolded protein response. Cell 2016;167:1867–1882.e21. 10.1016/j.cell.2016.11.048. 27984733 PMC5315571

[4] Adamson B , Norman TM, Jost M. et al. A multiplexed single-cell CRISPR screening platform enables systematic dissection of the unfolded protein response. Cell 2016;167:1867–1882.e21. 10.1016/j.cell.2016.11.048. 27984733 PMC5315571

[5] Hanna RE , Doench JG. Design and analysis of CRISPR–Cas experiments. Nat Biotechnol 2020;38:813–23. 10.1038/s41587-020-0490-7. 32284587

[6] Nakamura M , Gao Y, Dominguez AA. et al. CRISPR technologies for precise epigenome editing. Nat Cell Biol 2021;23:11–22. 10.1038/s41556-020-00620-7. 33420494

[7] Frangieh CJ , Melms JC, Thakore PI. et al. Multimodal pooled Perturb-CITE-seq screens in patient models define mechanisms of cancer immune evasion. Nat Genet 2021;53:332–41. 10.1038/s41588-021-00779-1. 33649592 PMC8376399

[8] Przybyla L , Gilbert LA. A new era in functional genomics screens. Nat Rev Genet 2022;23:89–103. 10.1038/s41576-021-00409-w. 34545248

[9] Stringer C , Wang T, Michaelos M. et al. Cellpose: a generalist algorithm for cellular segmentation. Nat Methods 2021;18:100–6. 10.1038/s41592-020-01018-x. 33318659

[10] Littman R , Hemminger Z, Foreman R. et al. Joint cell segmentation and cell type annotation for spatial transcriptomics. Mol Syst Biol 2021;17:e10108. 10.15252/msb.202010108. 34057817 PMC8166214

[11] Zeng Y , Zhou X, Rao J. et al. Accurately clustering single-cell RNA-seq data by capturing structural relations between cells through graph convolutional network. In: 2020 IEEE International Conference on Bioinformatics and Biomedicine (BIBM), pp. 519–22. Piscataway, NJ, USA: IEEE, 2020.

[12] Fan Z , Zhao H, Zhou J. et al. A versatile attention-based neural network for chemical perturbation analysis and its potential to aid surgical treatment: a experimental study. Int J Surg 2024;10–1097. 10.1097/JS9.0000000000001781. PMC11634177 39017949

[13] Hengshi Y , Welch JD. Perturbnet predicts single-cell responses to unseen chemical and genetic perturbations. BioRxiv 2022.07.20.500854. 10.1101/2022.07.20.500854. PMC12322087 40640612

[14] Mohammad Lotfollahi F , Wolf A, Theis FJ. Scgen predicts single-cell perturbation responses. Nat Methods 2019;16:715–21. 10.1038/s41592-019-0494-8. 31363220

[15] Sadria M , Layton A. The power of two: Integrating deep diffusion models and variational autoencoders for single-cell transcriptomics analysis. BioRxiv 2023.04.13.536789. 10.1101/2023.04.13.536789. PMC11927372 40119479

[16] Kingma DP , Welling M. Auto-encoding variational bayes. arXiv preprint arXiv:1312.6114. 2013. 10.48550/arXiv.1312.6114.

[17] Ho J , Jain A, Abbeel P. Denoising diffusion probabilistic models. Adv Neural Inf Process Syst 2020;33:6840–51.

[18] Wei X , Dong J, Wang F. scPreGAN, a deep generative model for predicting the response of single-cell expression to perturbation. Bioinformatics 2022;38:3377–84. 10.1093/bioinformatics/btac357. 35639705

[19] Goodfellow I , Pouget-Abadie J, Mirza M. et al. Generative adversarial networks. Commun ACM 2020;63:139–44. 10.1145/3422622.

[20] Yang Y , Li G, Zhong Y. et al. Gene knockout inference with variational graph autoencoder learning single-cell gene regulatory networks. Nucleic Acids Res 2023;51:6578–92. 10.1093/nar/gkad450. 37246643 PMC10359630

[21] Kamimoto K , Stringa B, Hoffmann CM. et al. Dissecting cell identity via network inference and in silico gene perturbation. Nature 2023;614:742–51. 10.1038/s41586-022-05688-9. 36755098 PMC9946838

[22] Aibar S , González-Blas CB, Moerman T. et al. SCENIC: single-cell regulatory network inference and clustering. Nat Methods 2017;14:1083–6. 10.1038/nmeth.4463. 28991892 PMC5937676

[23] Wu Y , Barton RA, Wang Z. et al. Predicting cellular responses with variational causal inference and refined relational information. arXiv preprint arXiv:2210.00116. 2022. 10.48550/arXiv.2210.00116.

[24] Roohani Y , Huang K, Leskovec J. Predicting transcriptional outcomes of novel multigene perturbations with gears. Nat Biotechnol 2024;42:927–35. 10.1038/s41587-023-01905-6. 37592036 PMC11180609

[25] Gong J , Hao M, Cheng X. et al. xTrimoGene: an efficient and scalable representation learner for single-cell RNA-seq data. Adv Neural Inf Process Syst 2024;36:1–7.

[26] Ruan J , Dean AK, Zhang W. A general co-expression network-based approach to gene expression analysis: comparison and applications. BMC Syst Biol 2010;4:1–21. 20122284 10.1186/1752-0509-4-8 PMC2829495

[27] Gene Ontology Consortium . The Gene Ontology (GO) database and informatics resource. Nucleic Acids Res 2004;32:258D–261. 10.1093/nar/gkh036. PMC308770 14681407

[28] Felix W , Souza A, Zhang T. et al. Simplifying graph convolutional networks. In: International conference on machine learning, pp. 6861–71. Cambridge, MA, USA: PMLR, 2019.

[29] Milacic M , Beavers D, Conley P. et al. The Reactome Pathway Knowledgebase 2024. Nucleic Acids Res 2024;52:D672–8. 10.1093/nar/gkad1025. 37941124 PMC10767911

[30] Replogle JM , Saunders RA, Pogson AN. et al. Mapping information-rich genotype-phenotype landscapes with genome-scale Perturb-seq. Cell 2022;185:2559–2575.e28. 10.1016/j.cell.2022.05.013. 35688146 PMC9380471

[31] Norman TM , Horlbeck MA, Replogle JM. et al. Exploring genetic interaction manifolds constructed from rich single-cell phenotypes. Science 2019;365:786–93. 10.1126/science.aax4438. 31395745 PMC6746554

[32] Hao M, Gong J, Zeng X. et al. Large-scale foundation model on single-cell transcriptomics. Nat Methods 2024;21:1481–91. 10.1038/s41592-024-02305-7. 38844628

[33] Bock C , Datlinger P, Chardon F. et al. High-content CRISPR screening. Nat Rev Methods Primers 2022;2:1–23. 10.1038/s43586-021-00093-4. PMC10200264 37214176

